# Cerebrospinal fluid Aβ42, t-tau, and p-tau levels in the differential diagnosis of idiopathic normal-pressure hydrocephalus: a systematic review and meta-analysis

**DOI:** 10.1186/s12987-017-0062-5

**Published:** 2017-05-10

**Authors:** Zhongyun Chen, Chunyan Liu, Jie Zhang, Norman Relkin, Yan Xing, Yanfeng Li

**Affiliations:** 1 Department of Neurology, Aviation General Hospital of China Medical University & Beijing Institute of Translational Medicine, Chinese Academy of Sciences, No. 3 Anwai Beiyuan Road, Chaoyang District, Beijing, 100012 China; 2 Department of Neurology and Neuroscience, Weill Medical College of Cornell University, Cornell Memory Disorders Program, 428 East 72 Street, Suite 500, New York, NY 10021 USA; 3 Department of Neurology, Peking Union Medical College Hospital, Beijing, China

**Keywords:** CSF biomarkers, Idiopathic normal-pressure hydrocephalus, Alzheimer’s disease, Meta-analysis, Systematic review

## Abstract

**Objectives:**

The purpose of this systematic review and meta-analysis was to evaluate the performance of cerebrospinal fluid (CSF) beta amyloid 42 (Aβ42), total tau (t-tau), and phosphorylated tau (p-tau) as potential diagnostic biomarkers for idiopathic normal-pressure hydrocephalus (iNPH) and to assess their utility indistinguishing patients with iNPH from those with Alzheimer disease (AD) and healthy normal controls.

**Methods:**

Studies were identified by searching PubMed, Embase, the Cochrane Library, Web of Science, Chinese National Knowledge Infrastructure (CNKI), Wanfang Chinese Periodical Database, VIP Chinese database, and Chinese Bio-medicine Database (CBM) before August 2016. The standardized mean difference (SMD) and 95% confidence interval (CI), comparing CSF Aβ42, t-tau, and p-tau levels between iNPH, AD and healthy controls, were calculated using random-effects models. Subgroup analyses were created according to ethnicity (Caucasian or Asian) and CSF type (lumbar or ventricular), and the publication bias was estimated using Egger’s test and the Begg’s test.

**Results:**

A total of 10 studies including 413 patients with iNPH, 186 patients with AD and 147 healthy controls were included in this systematic review and meta-analysis. The concentrations of CSF t-tau, and p-tau were significantly lower in iNPH patients compared to AD (SMD = −1.26, 95% CI −1.95 to −0.57, *P* = 0.0004; SMD = −1.54, 95% CI −2.34 to −0.74, *P* = 0.0002, respectively) and lower than healthy controls (SMD = −0.80, 95% CI −1.50 to −0.09, *P* = 0.03; SMD = −1.12, 95% CI −1.38 to −0.86, *P* < 0.00001, respectively). Patients with iNPH had significantly lower Aβ42 levels compared with controls (SMD = −1.14, 95% CI −1.74 to −0.55, *P* = 0.0002), and slightly higher Aβ42 levels compared with AD patients (SMD = 0.32, 95% CI 0.00–0.63, *P* = 0.05). Subgroup analyses showed that the outcomes may have been influenced by ethnicity and CSF source. Compared to AD, overall sensitivity in differentiating iNPH was 0.813 (95% CI 0.636–0.928) for Aβ42, 0.828 (95% CI 0.732–0.900) for t-tau, 0.943 (95% CI 0.871–0.981) for p-tau. Relative to AD, overall specificity in differentiating iNPH was 0.506 (95% CI 0.393–0.619) for Aβ42, 0.842 (95% CI 0.756–0.907) for t-tau, 0.851 (95% CI 0.767–0.914) for p-tau.

**Conclusion:**

The results of our meta-analysis suggest that iNPH may be associated with significantly reduced levels of CSF Aβ42, t-tau and p-tau compared to the healthy normal state. Compared to AD, both t-tau and p-tau were significantly decreased in iNPH, but CSF Aβ42 was slightly increased. Prospective studies are needed to further assess the clinical utility of these and other CSF biomarkers in assisting in the diagnosis of iNPH and differentiating it from AD and other neurodegenerative disorders.

**Electronic supplementary material:**

The online version of this article (doi:10.1186/s12987-017-0062-5) contains supplementary material, which is available to authorized users.

## Background

Normal pressure hydrocephalus (NPH) was first described in 1965 by Hakim, Adams and colleagues [1] as a syndrome of cerebral ventricular enlargement occurring in adults without elevated cerebrospinal fluid (CSF) pressure or macroscopic obstruction to CSF flow. Early studies identified NPH as a progressive but treatable disorder that often presents with the classical symptom triad of gait disturbance, dementia, and urinary incontinence. This condition is considered idiopathic NPH (iNPH) when there is no identifiable antecedent cause and secondary NPH (sNPH) when events such as severe head trauma, subarachnoid hemorrhage or meningitis precede its onset.

Recent studies have reported prevalence rates of iNPH ranging from 0.51 to 5.9% in the elderly population that increase with advancing age. This suggests that iNPH is much more common than previously recognized [2–4]. It is extremely underdiagnosed throughout most of the world [5] and less than 10–20% of patients with iNPH get appropriate specialized treatment [6, 7]. This is particularly unfortunate because iNPH and sNPH can be effectively treated by neurosurgical placement of a shunt, which leads to improvement or stabilization of symptoms in upwards of 80% of accurately diagnosed patients [8].

An important factor in the under-diagnosis of iNPH is that early clinical features may be subtle and its manifestations can overlap those of other neurological disorders such as AD and normal brain aging. Therefore, finding sensitive and specific tools for early and accurate differential diagnosis is vital for improving the detection and care of patients with iNPH.

Analysis of CSF biomarkers is performed as part of many neurodegenerative research studies and is increasingly employed in the clinical diagnostic work-up when neurodegenerative disorders are suspected. Aβ42, t-tau, and p-tau have been widely validated as CSF biomarkers for AD diagnosis. In particular, a pattern of reduced CSF Aβ42 with elevated CSF p-tau and t-tau is strongly associated with AD [9]. The Aβ42 protein is closely linked to AD pathology as the central component of extracellular neuritic plaques [10]. Tau is an intracellular microtubule-associated protein and its total level in CSF is thought to reflect the extent of ongoing neuronal death. Hyperphosphorylated forms of tau (p-tau) are more closely associated with neurofibrillary tangle formation in AD and measurement of CSF p-tau therefore adds specificity for AD to the CSF biomarker profile [10, 11].

Comparably fewer CSF biomarker studies have been performed on iNPH patients and there has been only moderate consistency among those performed. Some studies have reported that patients with iNPH have low Aβ42 similar to those in AD, but without increased t-tau and p-tau levels. There has been speculation that this pattern might be useful for differentiating iNPH from AD [12, 13]. However, other studies did not reach the same conclusion [14, 15]. Considering these inconsistent results, we conducted a meta-analysis to evaluate whether CSF Aβ42, t-tau, and p-tau levels are of value in the differential diagnosis of iNPH from AD and healthy normal controls.

## Methods

### Literature search

We did asystematic review and meta-analysis according to the PRISMA guidelines [16]. Two authors searched PubMed, Embase, the Cochrane Library, Web of Science, Chinese National Knowledge Infrastructure (CNKI), Wanfang Chinese Periodical Database, VIP Chinese database, and Chinese Bio-medicine Database (CBM) for relevant articles published before August 2016 by using Medical Subject Heading (MeSH) terms and the following free text terms: “((normal pressure OR normotensive) AND hydrocephal*) OR Hydrocephalus, Normal Pressure [Mesh]” AND “((biological) AND markers) OR biomarker OR CSF OR cerebrospinal” AND “Aβ42 OR Abeta42 OR Abeta-42 OR Aβ1-42 OR t-tau OR p-tau OR tau OR phospho-tau OR phosphorylated tau”.

The search was confined to human studies published in English and Chinese. The titles and abstracts of each article were scanned independently by two authors (ZYC and CYL) to exclude studies that were clearly irrelevant. The full text of the remaining studies were then retrieved and assessed for eligibility according to the inclusion criteria. Any disagreement was resolved by discussion with a third author (JZ).

### Study selection

Studies were eligible for the analysis if they fulfill the following criteria: (1) case–control studies which compared the CSF levels of Aβ42 and/or t-tau and/or p-tau between iNPH patients and AD patients or healthy controls; (2) clearly stated iNPH and AD diagnostic criteria (see Additional file 1: Table S1); (3) original articles containing independent data, and data were expressed as mean and standard deviation (SD) or median and interquartile range (IQR).

The exclusion criteria for the study were as follows: (1) abstracts, reviews, case reports, animal experiments, experts’ opinions and commentaries; (2) duplicate publications of the same dataset; (3) papers not specifically focused on iNPH (i.e.: NPH or sNPH).

### Data extraction

Two authors extracted data from the included articles, which included the following: the first author’s name, year of publication, country, number of cases and controls, age (mean ± SD or median and interquartile range), the number of females and males, the CSF levels of Aβ42, t-tau and p-tau concentration (mean ± SD or median and interquartile range), analytical technology, and CSF source.

### Quality evaluation

The Newcastle-Ottawa Quality Assessment Scale (NOS) was used to assess the quality of each included study, and was performed by two authors independently, with a third author consulted in case of discrepancy. Three major components were collected: (1) the selection (0–4 points); (2) the exposure (0–3 points); 3) the comparability (0–2 points). Higher scores represent better quality in methodology. All studies in this systematic review had scores greater than or equal to seven, indicating good qualities.

### Statistical analysis

Statistical analyses were performed using Review Manager 5.1.2 (Cochrane Collaboration, Oxford, UK), Meta DiSc 1.4 version (Cochrane Collaboration, Oxford, UK) and Stata 12.0 (Stata Corp, College Station, Texas, USA). Data given as median and IQR were converted into mean ± SD in accordance with the protocol provided by Wan et al. [17]. In studies where patients with iNPH were divided into shunt responder and shunt non-responders, we combined the two datasets for the purpose of the current evaluation [18].

The standardized mean difference (SMD) and the corresponding 95% CI were used as the main effect measure. Heterogeneity across the studies was estimated by using the Chi square based Cochran Q and the *I* ^2^ test statistics [19]. The heterogeneity was considered statistically significant if *P* < 0.1 or *I* ^*2*^ > 50%, a random-effects model (DerSimonian–Laird method) of analysis was used; otherwise, the fixed-effects model (Mantel–Haenszel method) was applied instead. Sub-groups were created according to ethnicity (Caucasian or Asian) and CSF source (lumbar or ventricular). Sensitivity analysis was performed by removing studies one by one to detect its influence on pooled ORs. The Egger’s test and the Begg’s test were used to estimate the severity of publication bias [20]. Where publication bias existed, we used the Trim and Fill method to correct it. The overall sensitivity, specificity, positive likelihood ratio (PLR), and negative likelihood ratio (NLR), as well as their corresponding 95% CIs, were pooled based on the random effects model. In addition, the area under the curve (AUC) and Q* index were calculated to evaluate the diagnostic test accuracy. All statistical tests were 2-sided, and statistical significance was defined as *P* < 0.05.

## Results

### Included studies

**Fig. 1:**
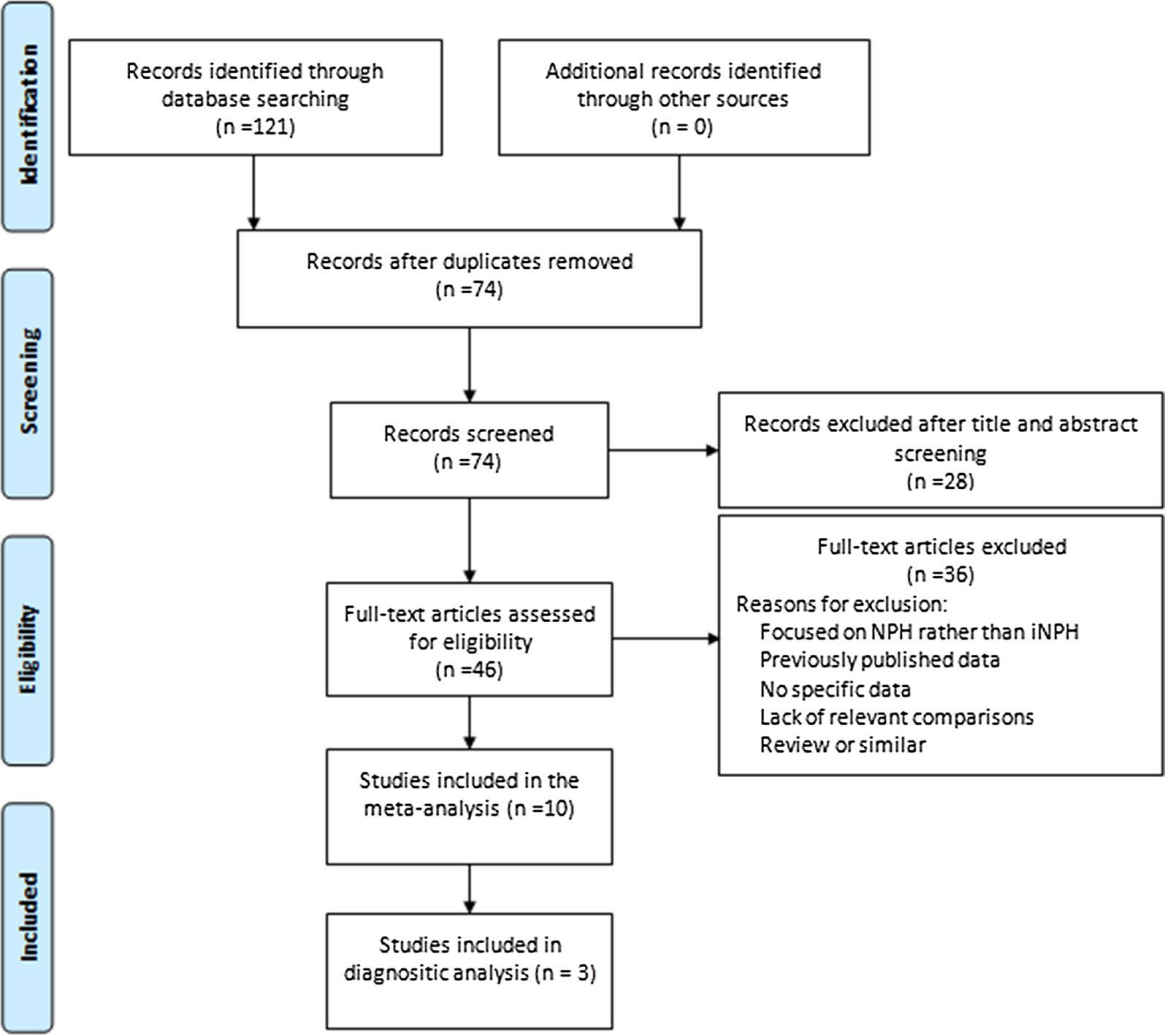
Flow chart of the search and selection process

A total of 121 relevant articles were identified in the initial search. Seventy-four articles remained after removal of duplicate studies and 28 articles were excluded based on titles and abstracts. After systematically reviewing the remaining 46 full-text articles, 36 articles were excluded for not fulfilling our inclusion criteria. Finally, ten articles met stringent search criteria for data analysis and three articles were included in the diagnostic analysis. A detailed flow chart of the search and selection process is depicted in Fig. 1.

### Study characteristics

The characteristics of the ten studies included in the meta-analysis are listed in Table 1. A total of 413 patients with iNPH, 186 patients with AD, and 147 healthy controls were included in this meta-analysis. 2 studies were performed in the United States [21, 22], 2 in Japan [23, 24], 2 in Sweden [12, 25], 1 in Greece [13], 1 in Finland [26], 1 in South Korea [27] and 1 in Italy [28]. These studies were published between 2007 and 2015. With respect to the assay method used to measure CSF Aβ42, t-tau, p-tau levels, 9 studies were performed using ELISA methodology and 1 was conducted using other methods.

**Table 1:** Characteristics of studies included in the meta-analysis

Study	Country	Patients	N	Age	Male	Aβ42 (pg/ml)	t-tau (pg/ml)	p-tau (pg/ml)	Method	CSF type
Agren-Wilsson et al. [12]	Sweden	iNPH	62	72 (55–83)^d^	39/23	503 ± 103	171 ± 68	33 ± 10	ELISA	Lumbar
		Controls^a^	23	73 (59–88)^d^	10/13	716 ± 170	330 ± 179	58 ± 29		
Kapaki et al. [13]	Greece	iNPH	18	69 ± 14	11/7	400 ± 219	219 (118–280)^d^	34.8 (20–46.7)^d^	ELISA	Lumbar
		AD	67	66 ± 10	26/41	422 ± 149	571 (443–984)^d^	72 (60.4–100)^d^		
		Controls^a^	72	64 ± 11	41/31	721 ± 228	166 (117–233)^d^	45.1 (40–55.8)^d^		
Seppala et al. [26]	Finland	iNPH	101	NA	NA	399 ± 188	1261 ± 1354	78.1 ± 51.5	ELISA	Ventricular
		AD	51	75.4 ± 7.6	27/24	360 ± 202	1432 ± 1990	83.9 ± 50.1		
Jeppsson et al. [25]	Sweden	iNPH	28	69 ± 6.6	15/13	221 (156–325)^d^	39 (34–50)^d^	39 (33–50)^d^	ECL^e^, xMAP	Lumbar
		Controls^a^	20	70 ± 3.6	11/9	498 (391–669)^d^	84 (64–107)^d^	59 (47–75)^d^		
Miyajima et al. [24]	Japan	iNPH	46	74.7 ± 6.9	27/19	314 ± 254	152 ± 98	24.2 ± 10.5	ELISA	Lumbar
		AD	10	78.4 ± 8.5	5/5	189 ± 116	536 ± 299	90.3 ± 32.2		
		Controls^a^	8	67.1 ± 6.7	2/6	255 ± 129	216 ± 138	31.6 ± 4.66		
Lim et al. [27]	South Korea	iNPH	25	73.3 ± 7.0	12/13	579.8 ± 182.3	131.9 ± 77.6	27.0 ± 9.6	ELISA	Lumbar
		AD	17	72.2 ± 10.0	10/7	409.2 ± 166.1	259.6 ± 161.5	51.3 ± 28.3		
		Controls^a^	10	63.0 ± 6.7	3/7	691.8 ± 212.7	196.9 ± 114.4	43.0 ± 28.5		
Pyykko et al. [21]	America	iNPH^b^	48	NA	27/26	476 ± 203	1210 ± 1186	77.1 ± 51.7	ELISA	Ventricular
		iNPH^c^	5	NA		428 ± 250	562 ± 443	50.4 ± 14.3		
		AD	16	78.3 (54.1–85.7)^d^	6/10	422 ± 259	1361 ± 1687	81.3 ± 51.2		
Tsai et al. [22]	America	iNPH	11	81.36 ± 2.58	2/9	251.17 ± 92.39	475.07 ± 335.23	47.35 ± 20.24	ELISA	Lumbar
		AD	11	61.46 ± 8.24	7/4	290.33 ± 146.74	651.53 ± 276.41	79.12 ± 30.00		
Schirinzi et al. [28]	Italy	iNPH	14	73.21 ± 4.63	8/6	477.50 ± 223.10	183.36 ± 99.81	25.36 ± 9.48	ELISA	Lumbar
		AD	14	69.85 ± 7.42	5/9	308.43 ± 91.38	662.79 ± 223.62	77.71 ± 21.65		
		Controls^a^	14	67.21 ± 7.67	8/6	862.86 ± 230.40	231.14 ± 78.42	40.50 ± 11.81		
Jingami et al. [23]	Japan	iNPH	55	76.4 (7.2)	NA	NA	297 (232–440)^d^	16.0 (11.3–23.7)^d^	ELISA	Lumbar
		AD	20	71.8 (12.8)	NA	NA	932 (716–1533)^d^	57.0 (32.1–102)^d^		

*NA* not available

^a^Healthy controls

^b^Shunt responder

^c^Shunt nonresponder

^d^Data are presented as median (Q1–Q3 range)

^e^Electrochemiluminescence

### Meta-analysis

#### Pooled analysis (Table 2)

##### Aβ42 in iNPH versus AD/healthy controls

Seven studies, including 268 patients with iNPH and 186 patients with AD and 6 studies, including 193 patients with iNPH and 147 healthy controls were used in the meta-analysis, respectively. A random-effect model was used to calculate pooled SMD because of highly significant heterogeneity among those studies (iNPH versus AD, *P* < 0.06, *I* ^2^ = 50%; iNPH versus healthy controls, *P* = 0.0003, *I* ^2^ = 79%). Patients with iNPH showed significantly decreased Aβ42 levels compared with healthy controls (SMD = −1.14, 95% CI −1.74 to −0.55, *P* = 0.0002), and slightly increased Aβ42 levels compared with AD patients (SMD = 0.32, 95% CI 0.00–0.63, *P* = 0.05).

**Table 2:** Pooled analysis and subgroup analysis of CSF Aβ42, t-tau, and p-tau between iNPH and AD/healthy controls

	Stratification group	iNPH vs. AD							iNPH vs. healthy controls						
		Comparisons			SMD [95% CI]	Heterogeneity test			Comparisons			SMD [95% CI]	Heterogeneity test		
		Studies (N_iNPH_/N_AD_)	Z	P		Q	P	I^2^, %	Studies (N_iNPH_/N_Control_)	Z	P		Q	P	I^2^, %
Aβ42	Total	7 (268/186)	1.98	0.05	0.32 (0.00, 0.63)	11.93	0.06	50	6 (193/147)	3.75	0.0002	−1.14 (−1.74, −0.55)	23.55	0.0003	79
	Study population														
	Caucasian	5 (197/159)	1.41	0.16	0.17 (−0.07, 0.40)	6.47	0.17	38	4 (122/129)	9.94	<0.00001	−1.60 (−1.91, −1.28)	0.67	0.88	0
	Asian	2 (71/27)	3.09	0.002	0.75 (0.27, 1.22)	0.79	0.37	0	2 (71/18)	0.41	0.68	−0.17 (−0.96, 0.63)	2.26	−0.13	56
	CSF source														
	Lumbar	5 (114/119)	1.49	0.14	0.39 (−0.12, 0.91)	11.41	0.02	65	6 (193/147)	3.75	0.0002	−1.14 (−1.74, −0.55)	23.55	0.0003	79
	Ventricular	2 (154/67)	1.41	0.16	0.21 (−0.08, 0.50)	0.00	0.95	5	–	–	–	–	–	–	–
t-tau	Total	8 (323/206)	3.57	0.0004	−1.26 (−1.95, −0.57)	70.50	<0.00001	90	6 (193/147)	2.22	0.03	−0.80 (−1.50, −0.09)	35.00	<0.00001	86
	Study population														
	Caucasian	5 (197/159)	2.30	0.02	−0.86 (−1.60, −0.13)	30.2	<0.00001	87	4 (122/129)	1.63	0.10	−0.87 (−1.91, 0.17)	34.81	<0.00001	91
	Asian	3 (126/47)	4.37	<0.00001	−1.88 (−2.72, −1.03)	8.69	0.01	77	2 (71/18)	2.41	0.02	−0.66 (−1.19, −0.12)	0.04	0.84	0
	CSF source														
	Lumbar	6 (169/139)	5.28	<0.00001	−1.66 (−2.28, −1.05)	21.18	0.0008	76	6 (193/147)	2.22	0.03	−0.80 (−1.50, −0.09)	35.00	<0.00001	86
	Ventricular	2 (154/67)	0.83	0.41	−0.12 (−0.41, 0.17)	0.03	0.87	0	–	–	–	–	–	–	–
p-tau	Total	8 (323/206)	3.78	0.0002	−1.54 (−2.34, −0.74)	89.64	<0.00001	92	6 (193/147)	8.36	<0.00001	−1.12 (−1.38, −0.86)	3.4	0.64	0
	Study population														
	Caucasian	5 (197/159)	2.49	0.01	−1.11 (−1.99, −0.24)	40.87	<0.00001	90	4 (122/129)	7.9	<0.00001	−1.21 (−1.51, −0.91)	1.82	0.61	0
	Asian	3 (126/47)	3.21	0.001	−2.23 (−3.59, −0.87)	20.55	<0.00001	90	2 (71/18)	2.99	0.003	−0.83 (−1.37, −0.29)	0.11	0.74	0
	CSF source														
	Lumbar	6 (169/139)	5.42	<0.00001	−2.03 (−2.77, −1.30)	27.56	<0.00001	82	6 (193/147)	8.36	<0.00001	−1.12 (−1.38, −0.86)	3.4	0.64	0
	Ventricular	2 (154/67)	0.80	0.42	−0.12 (−0.41, 0.17)	0.00	0.95	0	–	–	–	–	–	–	–

##### T-tau in iNPH versus AD/healthy controls

Eight studies reported values for CSF t-tau in 323 iNPH patients and 206 AD patients and 6 studies reported values for CSFt-tau in 193 iNPH patients and 147 healthy controls. A significant heterogeneity across studies was found (iNPH versus AD, *P* < 0.00001, *I* ^2^ = 90%; iNPH versus healthy controls, *P* < 0.00001, *I* ^2^ = 86%), thus the random-effects model was used to calculate the pooled SMD. T-tau levels were significantly lower in iNPH patients than in AD (SMD = −1.26, 95% CI −1.95 to −0.57, *P* = 0.0004) and significantly lower than in healthy controls (SMD = −0.80, 95% CI −1.50 to −0.09, *P* = 0.03).

##### P-tau in iNPH versus AD/healthy controls

Mean P-tau values of iNPH patients were compared with AD patients in 8 articles, including 323 iNPH patients and 206 AD patients. 6 studies reported CSF values for p-tau in 194 iNPH patients and 147 healthy controls. A significant heterogeneity across studies was found in iNPH versus AD (*P* < 0.00001, *I* ^2^ = 92%), while no heterogeneity was found between iNPH and healthy controls (*P* = 0.64, *I* ^2^ = 0%). P-tau levels were significantly lower in iNPH patients than in AD (SMD = −1.54, 95% CI −2.34 to −0.74, *P* = 0.0002) and significantly lower than in healthy controls (SMD = −1.12, 95% CI −1.38 to −0.86, *P* < 0.00001).

#### Subgroup analysis

A subgroup analysis was performed according to the categories of country (Asia or others) and CSF source (lumbar or ventricular) (see Table 2).

Of the ten studies included in this meta-analysis, 3 studies were performed in Asian populations, whereas 7 studies were performed in Caucasian groups. For Aβ42 levels in iNPH versus AD, the pooled SMD was 0.75 (95% CI 0.27–1.22, *P* = 0.002) in Asian groups and 0.17 (95% CI −0.07 to 0.40, *P* = 0.16) in Caucasian groups. For Aβ42 levels in iNPH versus healthy controls, the pooled SMD was −0.17 (95% CI −0.96 to 0.63, *P* = 0.58) in Asian groups and −1.60 (95% CI −1.91 to −1.28, *P* < 0.00001) in Caucasian groups. For t-tau levels in iNPH versus AD, the pooled SMD was −1.88 (95% CI −2.72 to −1.03, *P* < 0.00001) in Asian groups and −0.86 (95% CI −1.60 to −0.13, *P* = 0.02) in Caucasian groups. For t-tau levels in iNPH versus healthy controls, the pooled SMD was −0.66 (95% CI −1.19 to −0.12, *P* = 0.02) in Asian groups and −0.87 (95% CI −1.91 to 0.17, *P* = 0.10) in Caucasian groups. For p-tau levels in iNPH versus AD, the pooled SMD was −2.23 (95% CI −3.59 to −0.87, *P* = 0.001) in Asian groups and −1.11 (95% CI −1.99 to −0.24, *P* = 0.01) in Caucasian groups. For p-tau levels in iNPH versus healthy controls, the pooled SMD was −0.83 (95% CI −1.37 to −0.29, *P* = 0.003) in Asian groups and −1.21 (95% CI −1.51 to −0.91, *P* < 0.00001) in Caucasian groups.

Of the ten studies included in this meta-analysis, 8 studies were performed using lumbar CSF while 2 studies were using ventricular CSF. For Aβ42 levels in iNPH versus AD, the pooled SMD was 0.39 (95% CI −0.12 to 0.91, *P* = 0.14) in lumbar CSF groups and 0.21 (95% CI −0.08 to 0.50, *P* = 0.16) in ventricular CSF groups. For t-tau levels in iNPH versus AD, the pooled SMD was −1.66 (95% CI −2.28 to −1.05, *P* < 0.00001) in lumbar CSF groups and −0.12 (95% CI −0.41 to 0.17, *P* = 0.41) in ventricular CSF groups. For p-tau levels in iNPH versus AD, the pooled SMD was −2.03 (95% CI −2.77 to −1.30, *P* < 0.00001) in lumbar CSF groups and −0.12 (95% CI −0.41 to 0.17, *P* = 0.42) in ventricular CSF groups.

#### Sensitivity analysis

**Fig. 2:**
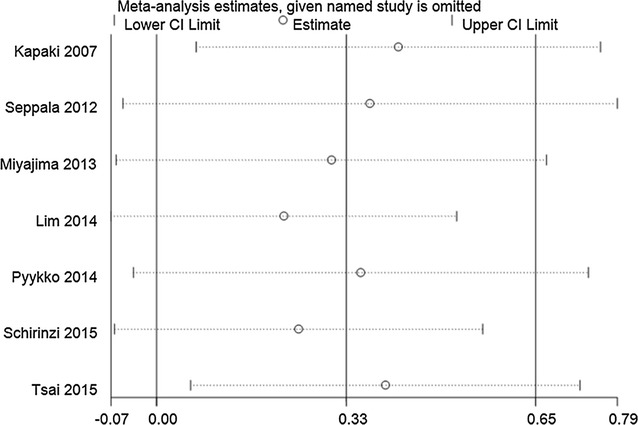
Sensitivity analysis of CSF Aβ42 levels in iNPH compared to AD. Sensitivity analysis was performed by removing studies one by one and comparing the pooled estimate from the remaining studies with the pooled estimate from all studies. The *X axis* represents the pooled estimate and the *Y axis* represents the excluded studies. Analyses were conducted on the raw data and the pooled estimate was 0.33 (95% CI 0.00–0.65). The pooled estimate was different when the study of Seppala et al. [26], Miyajima et al. [24], Lim et al. [27], Pyykko et al. [21], Schirinzi et al. [28] were omitted separately. The pooled estimates (95% CI) were 0.37 (95% CI −0.05 to 0.79), 0.30 (95% CI −0.07 to 0.67), 0.22 (95% CI −0.07 to 0.51), 0.35 (95% CI −0.04 to 0.74), 0.25 (95% CI −0.07 to 0.56), respectively. This suggests that results of those between-groups analyses were not stable and reliable

**Fig. 3:**
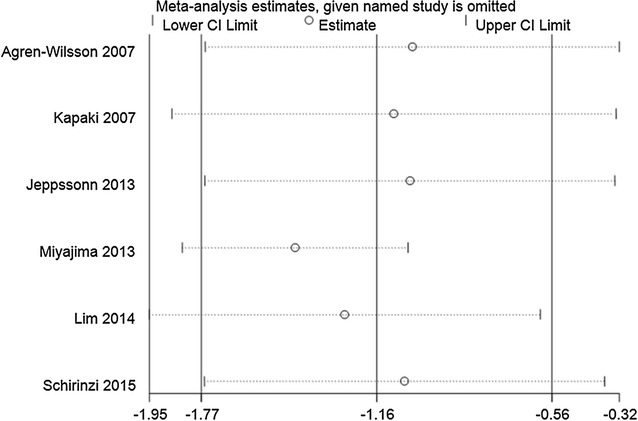
Sensitivity analysis of CSF Aβ42 levels in iNPH compared to healthy controls. Analyses were conducted on the raw data and the pooled estimate was −1.16 (95% CI −1.77 to −0.56). The direction and magnitude of pooled estimates did not change significantly after removing studies one by one, indicating that the results of the meta-analysis were relatively robust

**Fig. 4:**
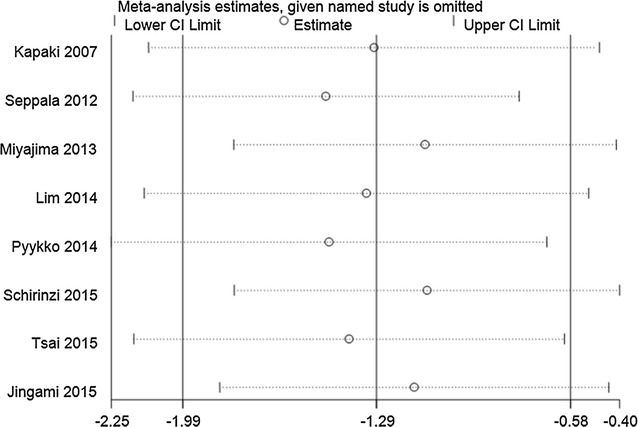
Sensitivity analysis of CSF t-tau levels in iNPH compared to AD. Analyses were conducted on the raw data and the pooled estimate was −1.29 (95% CI −1.99 to −0.58). The direction and magnitude of pooled estimates did not change significantly after removing studies one by one, indicating that the results of the meta-analysis were relatively robust

**Fig. 5:**
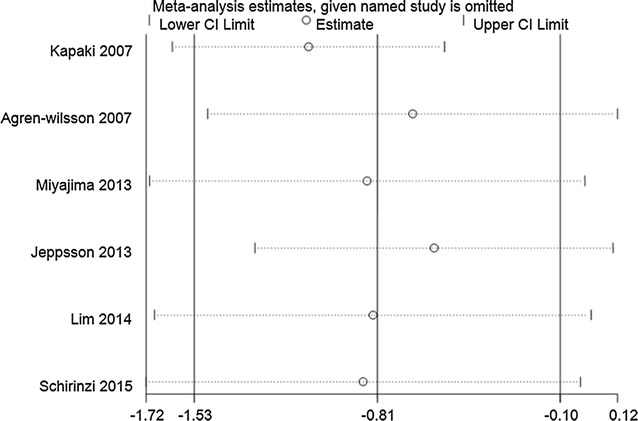
Sensitivity analysis of CSF t-tau levels in iNPH compared to healthy controls. Analyses were conducted on the raw data and the pooled estimate was −0.81 (95% CI −1.53 to −0.10). The pooled estimate was different when the study of Agren-Wilsson et al. [12], Jeppsson et al. [25], Lim et al. [27] were omitted separately. The pooled estimates (95% CI) were −0.68 (95% CI −1.47 to 0.12), −0.59 (95% CI −1.29 to 0.11) and −0.83 (95% CI −1.68 to 0.21), respectively. This suggests that results of those between-groups analyses were not stable and reliable

**Fig. 6:**
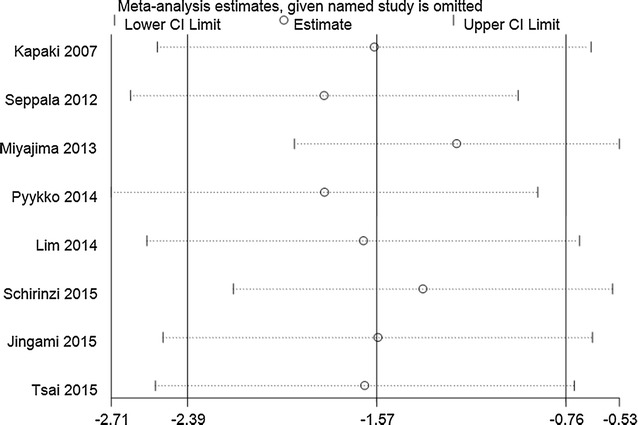
Sensitivity analysis of CSF p-tau levels in iNPH compared to AD. Analyses were conducted on the raw data and the pooled estimate was −1.57 (95% CI −2.39 to −0.76). The direction and magnitude of pooled estimates did not change significantly after removing studies one by one, indicating that the results of the meta-analysis were relatively robust

**Fig. 7:**
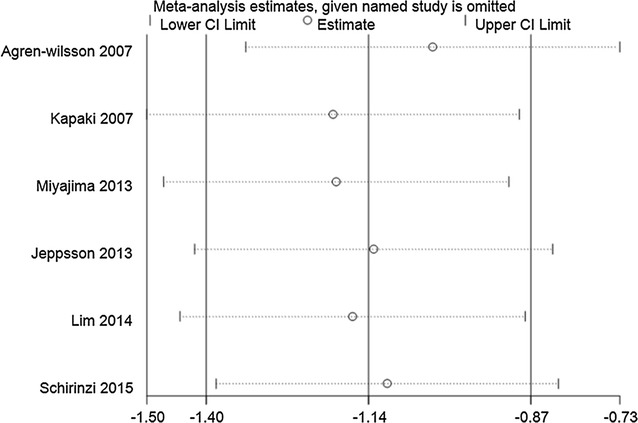
Sensitivity analysis of CSF p-tau levels in iNPH compared to healthy controls. Analyses were conducted on the raw data and the pooled estimate was −1.14 (95% CI −1.40 to −0.87). The direction and magnitude of pooled estimates did not change significantly after removing studies one by one, indicating that the results of the meta-analysis were relatively robust

Sensitivity analysis was performed by removing studies one by one and comparing the pooled estimate from the remaining studies with the pooled estimate from all studies. Sensitivity analysis of Aβ42 levels between iNPH and healthy controls, t-tau levels between iNPH and AD, and p-tau levels between iNPH and AD/healthy controls revealed that the direction and magnitude of pooled estimates did not change significantly, indicating that the results of the meta-analysis were relatively robust. In contrast, sensitivity analysis of Aβ42 levels in iNPH and AD, as well as t-tau levels in iNPH and healthy controls revealed that the pooled estimates were different when the leave-one-out approach was used. This suggests that results of those between-groups analyses were not stable and reliable (see Figs. 2, 3, 4, 5, 6, 7).

#### Publication bias

Begg and Egger tests were performed to assess for publication bias of the included studies and provide statistical evidence of publication funnel plot symmetry. Results showed that no significant publication bias was found in CSF Aβ42 levels between iNPH and AD (Begg’s test: Z = 0.60, P = 0.548; Egger’s test: t = 0.73, P = 0.500), Aβ42 levels between iNPH and healthy controls (Begg’s test: Z = 1.13, P = 0.260; Egger’s test: t = 0.96, P = 0.390), t-tau levels between iNPH and healthy controls (Begg’s test: Z = 0.00, P = 1.000; Egger’s test: t = −0.44, P = 0.686), p-tau levels between iNPH and healthy controls (Begg’s test: Z = 0.00, P = 1.000; Egger’s test: t = 0.55, P = 0.609). However evidence of publication bias was found in CSF t-tau levels between iNPH and AD (Begg’s test: Z = 0.87, P = 0.386; Egger’s test: t = −2.77, P = 0.032) and p-tau levels between iNPH and AD (Begg’s test: Z = 1.86, P = 0.063; Egger’s test: t = −3.69, P = 0.010). We therefore used the Trim and Fill method to correct it. There was no significant change in the results after using the trim and fill method, which suggested that the influence of publication bias on stability of results was weak.

#### Diagnostic results of included studies

Detailed data regarding the sensitivity, specificity and other diagnostic results were presented in Table 3. Compared to AD, higher Aβ42 concentrations differentiated iNPH with a sensitivity of 0.813 (95% CI 0.636–0.928) and a specificity of 0.506 (95% CI 0.393–0.619). The PLR and NLR of CSF Aβ42 concentrations in differentiating iNPH from AD were 2.032 (95% CI 0.918–4.498) and 0.324 (95% CI 0.156–0.673), respectively.

**Table 3:** Summary of the diagnostic results of the included studies

Study	Group compared	Cutoff value	Auc	Sensitivity	Specificity
Jingami et al. [23]	iNPH vs. AD				
	t-tau	766	0.9	75	98
	p-tau	24.4	0.91	95	74
Kapaki et al. [13]	iNPH vs. AD				
	Aβ42	268	0.58	90.9	44.4
	t-tau	294	0.84	92.5	77.8
	p-tau	47.4	0.83	88.7	86.7
Schirinzi et al. [28]	iNPH vs. AD				
	Aβ42	371	0.75	73.3	81.3
	t-tau	386	0.99	100	93.8
	p-tau	46	0.99	100	93.8

Relative to AD, the sensitivity and specificity of lower CSF t-tau concentrations in differentiating iNPH were 0.828 (95% CI 0.732–0.900) and 0.842 (95% CI 0.756–0.907), respectively. The PLR and NLR of CSF t-tau concentrations in differentiating iNPH were 8.199 (95% CI 1.738–38.678) and 0.112 (95% CI 0.018–0.699), respectively. The SROC AUC value was 0.963 ± 0.021, and the pooled diagnostic accuracy (Q*) was 0.909 ± 0.032.

Compared with AD, the sensitivity and specificity of lower CSF p-tau concentrations in distinguishing iNPH were 0.943 (95% CI 0.871–0.981) and 0.851 (95% CI 0.767–0.914), respectively. The PLR and NLR of CSF p-tau concentrations in distinguishing iNPH were 5.577 (95% CI 3.513–8.854) and 0.085 (95% CI 0.038–0.193), respectively. The SROC AUC value was 0.9453 ± 0.037, and the pooled diagnostic accuracy (Q*) was 0.884 ± 0.048.

## Discussion

In this systematic review and meta-analysis, we explored whether concentrations of CSF Aβ42, t-tau, and p-tau are of potential value in differentiating iNPH from AD and from healthy normal controls. Our results suggest that concentrations of CSF t-tau and p-tau in iNPH patients are lower than in AD patients and lower than healthy controls. Concentrations of Aβ42 in iNPH patients are lower than in healthy controls but slightly higher than in AD patients. Lower CSF t-tau and p-tau levels appear to carry higher sensitivity and specificity in differentiating iNPH from AD.

The combined pattern of reduced CSF levels of Aβ42 and increased levels of CSF t-tau and p-tau is an established CSF biomarker for AD [9]. The low Aβ42 levels compared to healthy controls in AD are believed to result from sequestration of soluble beta amyloid in plaques, while the elevated concentrations of t-tau and p-tau are thought to reflect release from the intraneuronal compartment owing to nerve cell and neurite damage. Past studies have fairly consistently found iNPH patients have low CSF Aβ42 levels in a range that overlaps that of AD. In this meta-analysis, we found that high Aβ42 levels in CSF might be slightly helpful in differentiating iNPH from AD, whereas low CSF Aβ42 could potentially be useful as a marker for differentiating of iNPH from healthy normal elderly.

Various hypotheses have been proposed to explain the reduction of CSF Aβ42 levels in iNPH patients. One hypothesis is that delivery of Aβ42 to the CSF compartment is impaired as a consequence of reduced centripetal flow of extracellular fluid in the brain caused by the retrograde CSF flow dynamics in iNPH [14, 25]. Xie et al. [29] found that Aβ clearance from the extracellular fluid is increased during sleep, as the interstitial space increases 60% in size at this time. Hence, Graff-Radford et al. [14] hypothesized that reduced CSF Aβ42 levels in iNPH may be related to the smaller extracellular space and having less room for the convective flux of CSF and interstitial fluid during sleep. The increased CSF levels of many proteins obtained from lumbar CSF drainage in iNPH provides indirect support for this theory. However, Hladky and Barrand [30] raised doubts about this theory and proposed a hypothesis that the elimination of Abeta from the brain might be associated with the perivascular lymphatic drainage pathways. Another possible explanation is that hypometabolism in the periventricular zone, as sometime seen on PET and SPECT studies in iNPH patients, may play a role in lowering generation of CSF Aβ42 [25].

Contrary to the consistency of finding low Aβ42 level in iNPH, there is only moderate agreement regarding the concentrations of CSF t-tau and p-tau. In 2007, Kapaki et al. [13] found that t-tau was slightly increased in iNPH and obviously increased in AD compared to healthy controls, while p-tau levels were significantly increased only in AD. Therefore, the authors concluded that CSF p-tau alone or in combination with t-tau may be a useful marker in the differentiation of iNPH from AD. However, other studies found that both t-tau and p-tau concentrations were significantly reduced in iNPH patients compared to AD [23] and healthy controls [25]. Our results suggest that concentrations of CSF t-tau and p-tau in iNPH are lower than in AD and healthy controls and this difference might be used to differentiate iNPH from AD or healthy controls. Prior reports have found that the concentrations of t-tau and p-tau increased with age [31, 32]. Since the average ages of iNPH patients were older than the AD patients in this study, one might have expected to find higher t-tau and p-tau levels in the iNPH group. On the contrary, we found the opposite. Hence, the differences in the concentrations of t-tau and p-tau observed between iNPH and AD are more likely associated with the pathophysiology of iNPH than age differences [31]. Reduced clearance from extracellular fluid and decreased brain metabolism of periventricular zone in iNPH may contribute to this phenomenon of reduction in CSF t-tau and p-tau levels in iNPH [14].

Prior studies reported that the ventricular CSF t-tau and p-tau levels are higher than in lumbar CSF samples [26]. In the studies we analyzed, there was marked heterogeneity in CSF levels depending on the location from which the CSF was collected. Therefore we performed subgroup analysis taking into account the CSF sources (lumbar CSF vs. ventricular CSF). Results showed that the lumbar CSF t-tau and p-tau levels in iNPH were lower than in AD, while no differences were found in ventricular CSF samples. This finding may support the hypothesis that lower CSF levels of proteins in iNPH relate to the disturbed circulation of CSF to the lumbar region in this disorder. Other possible explanations for this discrepancy include insufficient sample size or methodologic differences such as neuronal injury associated with placing a ventricular catheter which may elevate ventricular CSF tau and p-tau levels [12]. A subgroup analysis was also performed according to the categories of country (Asia vs. Caucasian). Results showed that the CSF Aβ42 and t-tau levels were distinct in different races, which suggested that outcomes may have been influenced by ethnicity. However, insufficient sample size or methodologic differences may also explain this contrast.

Our systematic review and meta-analysis has some limitations that should be acknowledged. First, only a limited number of studies were eligible for inclusion, particularly in the diagnostic analyses, which reduced the power of our meta-analysis. Secondly, we observed marked heterogeneity suggesting that there was a significant difference among the included studies, which can be attributed to variation in sample size, age, ethnicity, CSF source, different metrics (means and medians), and methodologic differences in sample collection and the assays employed. Although random-effects and subgroup analyses were performed, these parameters could not completely explain the heterogeneity. Other factors can be identified that may have contributed to the heterogeneity across studies. We were unable to assess what stage of iNPH (mild, moderate, severe) was included in the respective studies, and this could introduce some of the variance in the CSF results. In addition, we assume that the reported cases were pure iNPH or pure AD, but it is known that these conditions can occur together in many cases. The possible coincident occurrence of iNPH with AD or complications due to other diseases, could not be evaluated because these factors were not reported in the included articles. This is likely to be an important contributing factor in the heterogeneity [15, 27, 33]. Thirdly, sensitivity analysis of Aβ42 levels between iNPH and AD, t-tau levels between iNPH and healthy controls indicated that the meta-analysis for these variables had poor reliability. Fourthly, we did not include unpublished and non-peer reviewed studies and our analysis was restricted to publications in English and Chinese, creating the possibility geographic source biases. Finally, a weak publication bias was found in relation to reports of CSF t-tau levels between iNPH and AD, and p-tau levels between iNPH and AD. However, there was no significant change in the results after using the Trim and Fill method, which suggests that the influence of publication bias on stability of results was weak.

## Conclusion

Our study suggests that reduced CSF t-tau and p-tau maybe come useful markers for the differentiation of iNPH from AD or healthy controls. In addition, low Aβ42 levels contribute to distinguish iNPH and AD from healthy controls while Aβ42 levels are statistically slightly higher in iNPH compared to AD. Given the relative paucity of studies included and other limitations in this study, it is reasonable to assume our results should be interpreted with caution. In future, well-designed, large-scale prospective studies with well-controlled, standardization of experimental protocols for CSF biomarker measurements are warranted as a step towards improving the diagnosis and differential diagnosis of iNPH in clinical practice.

## Additional file

**Additional file 1.** Supplement characteristics of studies included in the meta-analysis.
