# Supplementary material for: Cerebrospinal fluid Aβ42, t-tau, and p-tau levels in the differential diagnosis of idiopathic normal-pressure hydrocephalus: a systematic review and meta-analysis
Source: Fluids Barriers CNS. 2017 May 10;14:13. doi: 10.1186/s12987-017-0062-5 (PMC5424383; doi:10.1186/s12987-017-0062-5)
Supplement: Supplementary file 1 — Additional file 1. Supplement characteristics of studies included in the meta-analysis. [file 12987_2017_62_MOESM1_ESM.doc]

**Table 1  Supplement characteristics of studies included in the meta-analysis**

| Study | Country | Patients | diagnostic criteria | shunt | N_response_/N_all_ |
| --- | --- | --- | --- | --- | --- |
| Agren-Wilsson et al., 2007 | Sweden | iNPH | clinical manifestation and CT/MRI imaging | Yes | 33/55 (60%) |
|  |  | Controls | Without known psychiatric or neurological disorder, undergoing hip or knee replacement surgery |  |  |
| Kapaki et al., 2007 | Greece | iNPH | clinical manifestation and CT/MRI imaging | No | - |
|  |  | AD | NINCDSADRDA^1^ Criteria |  |  |
|  |  | Controls | With no evidence of cognitive decline that underwent hernia repair or other minor surgery under spinal anesthesia but they were otherwise physically and mentally healthy. |  |  |
| Seppala et al. , 2012 | Finland | iNPH | According to the abnormal ICP findings indicating a shunt; no  known cause for secondary NPH; and no clinical AD in the end  of follow-up | Yes | 88/101 (87%) |
|  |  | AD | NINCDSADRDA^1^ and DSM-IV criteria |  |  |
| Jeppsson et al., 2013 | Sweden | iNPH | iNPH Guidelines^2^ | Yes | 20/27 (74%) |
|  |  | Controls | Controls were recruited from the population registry of the City of Gothenburg and the Swedish retired people’s organizatio |  |  |
| Miyajima et al., 2013 | Japan | iNPH | Japanese guideline criteria for probable iNPH^3^ | Yes | 26/46 (74%) |
|  |  | AD | NINCDSADRDA^1^ Criteria |  |  |
|  |  | Controls | without subjective cognitive impairment or known brain disease, with an MMSE score of >25 |  |  |
| Lim et al., 2014 | South Korea | iNPH | clinical manifestation and CT/MRI imaging and response to the lumbar puncture | No | - |
|  |  | AD | NINCDSADRDA^1^ Criteria |  |  |
|  |  | Controls | All normal control subjects scored in each cognitive domain test higher than the cutoff value |  |  |
| Pyykko et al., 2014 | America | iNPH | Possible iNPH included a clinical examination, CT or MRI scan, and 24 h intraventricular ICP monitoring together with a frontal cortical brain biopsy | Yes | 48/53 (91%) |
|  |  |  |  |  |  |
|  |  | AD | NINCDSADRDA^1^ Criteria |  |  |
| Schirinzi et al., 2015 | Italy | iNPH | iNPH guideline criteria for possible iNPH^2^ | No | - |
|  |  | AD | NINCDSADRDA^1^ Criteria |  |  |
|  |  | Controls | Without evidence of other neurodegenerative disorders, undergoing LP for suspected chronic polyneuropathy |  |  |
| Tsai et al., 2015 | America | iNPH | iNPH guideline criteria for iNPH^2^ | No | - |
|  |  | AD | NINCDSADRDA^1^ Criteria |  |  |
| Jingami et al., 2015 | Japan | iNPH | iNPH guideline criteria for probable iNPH^2^ | No | - |
|  |  | AD | NINCDSADRDA^1^ Criteria |  |  |

1 National Institute of Neurological and Communicative Diseases and Stroke Alzheimer’s Disease and Related Disorders Association (NINCDSADRDA).

2 Relkin N, Marmarou A, Klinge P, Bergsneider M, Black PM. Diagnosing idiopathic normal-pressure hydrocephalus. Neurosurgery 2005;57(3 suppl):S4–S16; discussion ii–v.

3 Ishikawa M, Hashimoto M, Kuwana N, et al. Guidelines for management of idiopathic normal pressure hydrocephalus. Neurol Med Chir (Tokyo) 2008; 48: Suppl.
